# Symptomatic Bradycardia Manifesting as Acute Hypothyroidism Following COVID-19 Infection: A Case Report

**DOI:** 10.7759/cureus.27533

**Published:** 2022-07-31

**Authors:** Jaydip Desai, Arsh N Patel, Courtney L Evans, Molly Triggs, Fulton Defour

**Affiliations:** 1 Department of Research, Alabama College of Osteopathic Medicine, Dothan, USA; 2 Internal Medicine, Thomas Hospital, Fairhope, USA

**Keywords:** infectious disease, internal medicine, thyroid, endocrine, covid 19

## Abstract

The role of the severe acute respiratory syndrome coronavirus 2 (SARS-CoV-2) virus and associated autoimmune phenomenon behind pathology development has been a scientific mystery since the onset of the pandemic in 2020. Early on, scientific studies showed coronavirus disease 2019 (COVID-19) being linked to many pathological consequences including blood clots, neurocognitive dysfunction, and cardiomyopathy. We present a case of acute hypothyroidism in an 88-year-old female with no previous history of thyroid dysfunction or disease. The eventual workup revealed a thyroid-stimulating hormone (TSH) of greater than 100,000 milli-international units per liter (mlU/L) and a thyroxine (free T4) level of less than 0.10 nanograms per deciliter (ng/dl). At the time of presentation, she was found to have a positive COVID-19 test despite being vaccinated. She was started on a levothyroxine injection, which led to eventual symptom resolution. Our aim of this case report is to highlight the possibility of her acute hypothyroidism being triggered by the onset of COVID-19.

## Introduction

Thyroid disorders are prevalent among 18.57% of aging women. Hypothyroid-associated symptoms are generally non-specific and can mimic the effects of natural aging. Prompt diagnosis and treatment are crucial to prevent detrimental outcomes such as myxedema coma or hypothyroid cardiomyopathy [1]. Immunological mechanisms for viral infections, such as severe acute respiratory syndrome coronavirus 2 (SARS-CoV-2), can lead to increased Th1 and Th17 immune system hyperactivity. As such, this association has been considered for the development of chronic lymphocytic thyroiditis with progression to primary thyroid insufficiency [2]. Pathophysiology of SARS-CoV-2 infection requires host-cell entry via the angiotensin-converting enzyme 2 (ACE2) receptor. It has been found that the ACE2 receptor has a higher concentration of receptors in the thyroid compared to the lung. Thyroiditis includes various forms of thyroid inflammation and has a wide range of presenting causes and characteristics. Environmental factors implicated in the presentation of subacute and autoimmune thyroiditis include viral origins [3]. This further supports inquiry into possible relationships between the viral infection onset and increased risk of viral subacute thyroiditis or autoimmune thyroiditis [4]. 

The hallmark of clinical evaluation in cases with hypothyroidism begins with a thorough history and physical exam raising suspicion for further diagnostic evaluation. Clinical features usually include gastrointestinal, cardiovascular, neurological, musculoskeletal, or reproductive symptoms. A major complication of hypothyroidism includes detrimental cardiovascular effects such as decreased cardiac output and increased systemic vascular resistance, mimicking the physiology of cardiomyopathy [5]. Diagnostically, thyroid-stimulating hormone (TSH) and free T4 levels are used for the initial evaluation of thyroid function status. In the following two to three months, it is recommended to repeat the initial labs, as well as the inclusion of thyroid peroxidase (TPO) antibodies screening to determine autoimmune causation. With the aforementioned cardiovascular complications, it becomes part of the clinical workup to mitigate any concurrent risk factors that may compromise cardiovascular functioning such as atherosclerotic disease or cardiomyopathy [5]. Current medical management suggested by the American Thyroid Association and American Association of Clinical Endocrinology recommends starting patients on levothyroxine therapy if any one of the following criteria are met: TSH levels greater than 10 milli-international unit per liter (mIU/L), presence of hypothyroid symptoms, presence of cardiovascular risk factors, or positive TPO antibodies [5].

Early diagnosis and proactive treatment measures provide the essentials in promoting positive patient outcomes in clinical cases of hypothyroidism. We present an elderly female who presented initially with clinical symptoms of bradycardia. After further diagnostic work-up, the patient was positive for SARS-CoV-2 coronavirus and had laboratory values correlating to hypothyroidism. Levothyroxine treatment as the standard management of hypothyroidism led to an eventual resolution of symptoms. It was later believed that her clinical onset of hypothyroidism was attributed to her concurrent infection of SARS-CoV-2 coronavirus. Our case report aims to outline the patient's clinical presentation and hospitalization course followed by a discussion investigating an etiologic link between SARS-CoV-2 coronavirus and acute-onset hypothyroidism.

## Case presentation

An 88-year-old female presented to the emergency department with a four-day history of bradycardia, orthopnea, weakness, and nausea. The patient noted that a heart rate of 40 beats per minute had been observed through the prior night, while confirming a prior history of bradycardia. She denied worsening pain or fevers. Review of systems was positive for fatigue, appetite loss, and leg swelling. Past medical history is significant for chronic kidney disease stage III, deep vein thrombosis, gastroesophageal reflux disease, hypertension, and squamous cell carcinoma (SCC) of the skin. She reported that she had previously taken Keytruda (Merck & Company, Rahway, NJ, USA) for her SCC, but discontinued the medication after developing Steven Johnson Syndrome with the second dose. Recent surgical history included a skin biopsy two weeks back. Her family history was significant for unspecified heart disease in her brother, father, and a cerebrovascular accident on her mother’s side. She denied any use of tobacco products, consumption of alcohol, or illegal substance use.

**Figure 1:**
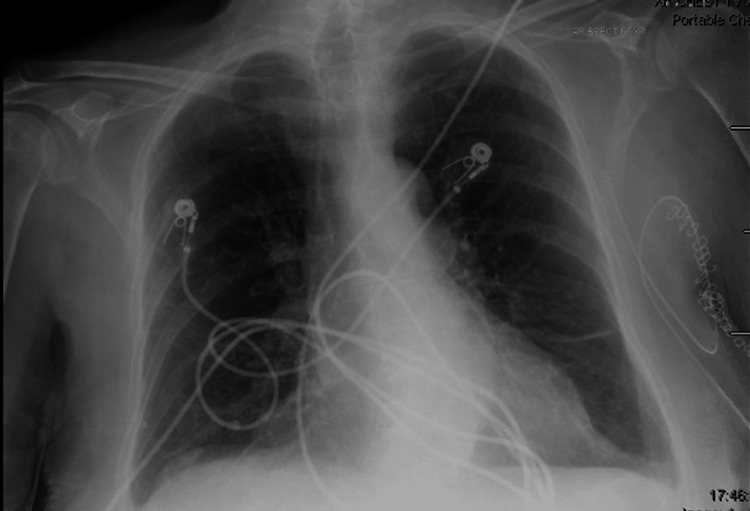
Chest X-ray taken on initial presentation in the Emergency Room. There was no evidence of cardiomegaly, interstitial or lobar infiltrates, cephalization of pulmonary vessels, or other findings consistent with heart failure.

On arrival to the Emergency Department (ED), vitals included heart rate of 64, oxygen saturation (SpO2) 98% on room air, temperature of 98.1, and blood pressure of 143/70. The physical exam was remarkable for distant heart sounds, distended abdomen without rebound or guarding and bilateral lower extremity edema extended to her calves. She was alert and oriented to person and place. Chest X-ray (CXR) demonstrated no infiltrates, hyperinflation, or cardiomegaly (Figure 1).

**Figure 2:**
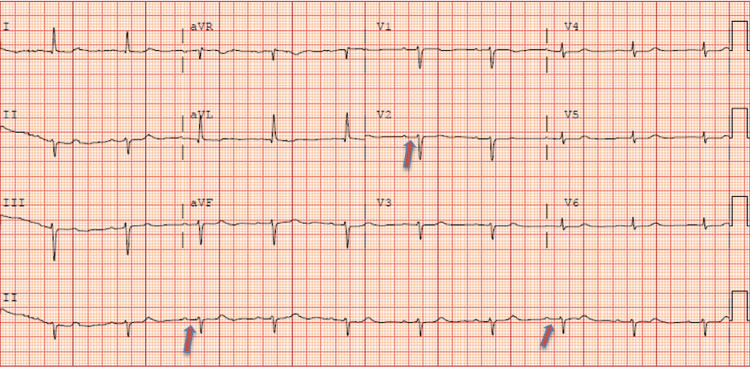
12-lead EKG showing bradycardia with first-degree AV block. The arrows represent lengthened PR intervals, consistent with a delay in conduction through the atrioventricular (AV) node.

Interpretation of 12-lead electrocardiogram (EKG) was remarkable for bradycardia with first-degree atrioventricular (AV) block (Figure 2). 

Complete blood count (CBC), complete metabolic profile (CMP), cardiac profile, and pro-brain natriuretic peptide studies were ordered for further evaluation (Table 1). Her TSH was 100,000 mIU/L. COVID-19 polymerase chain reaction (PCR) test was positive. The patient did not have any history of hypothyroidism before this presentation. She was started on levothyroxine 50 microgram (mcg) injections and was admitted inpatient for further management. On hospital admission day one, a Holter monitor ordered did not reveal any significant abnormalities. The patient already received two doses of the Pfizer COVID-19 vaccination, she was discharged after her quarantine. 

**Table 1:** Routine lab results obtained upon admission WBC: White Blood Cell; HGB: Hemoglobin; HCT: Hematocrit; PLT: Platelets; MCV: Mean Corpuscular Volume; MCH: Mean Corpuscular Hemoglobin; MCHC: Mean Corpuscular Hemoglobin Concentration; RDW: Red Cell Distribution Width; NA: Sodium; K: Potassium; CL: Chloride; CO2: Carbon Dioxide; BUN: Blood Urea Nitrogen; CK: Creatinine Kinase; TSH: Thyroid Stimulating Hormone; Free T4: Thyroxine; NT-PRO BNP: Brain Natriuretic Peptide; AST: Aspartate Aminotransferase; A/G Ratio: Albumin/Globulin ratio; ALT: Alanine Aminotransferase; GFR: Glomerular Filtration Ratio

Lab Finding	Reference Range	Value on Admission	Priority Level
WBC	4.5-12.0 K/uL	5.3	WNL
HGB	11.5-16.0 g/dL	9.9	LOW
HCT	35.0-48.0%	30%	LOW
PLT	120-450 K/uL	245	WNL
MCV	85-100 fl	93	WNL
MCH	27-33 pcg	31	WNL
MCHC	31-36 g/dL	33	WNL
RDW	0-14%	16	WNL
GLUCOSE	70-99 mg/mL	112	HIGH
SODIUM	135/145 mmol/L	135	WNL
POTASSIUM	3.5-5.1 mmol/L	3.8	WNL
CHLORIDE	98-107 mmol/L	97	WNL
ALBUMIN	3.5-5 g/dl	3.4	LOW
CO2	21-32 mmol/L	26	WNL
BUN	5-25 mg/dL	11	WNL
CREATININE	0.51-0.95 mg/dL	1.61	HIGH
CK TOTAL	26-192 U/L	213	HIGH
CK-MB	0-3.6 ng/mL	6.3	HIGH
TROPONIN-T	0-0.01 ng/mL	0.29	HIGH PANIC
TSH	0.358-7.740 mIU/L	>100,000	HIGH
FREE T4	0.93-1.70 ng/dL	<0.10	WNL
NT-PRO BNP	0-450 pg/mL	587	WNL
MAGNESIUM	1.6-2.6 mg/dL	2.6	WNL
CALCIUM	8.5-10.5mmol/L	7.9	LOW
TOTAL BILIRUBIN	0.2-1.0 mg/dL	0.3	WNL
ANION GAP	5-15 mEq/L	11	WNL
OSMOLALITY	267-291 mOsm/kg	270	WNL
AST	0-40u/L	74	HIGH
ALT	0-40u/L	27	WNL
TOTAL PROTEIN	6.4-8.2g/dL	5.5	WNL
GLOBULIN	2.0-4.2g/dL	2.1	LOW
A/G RATIO	1.00-2.20 RATIO	1.62	WNL
ALK PHOS	30-115u/L	123	HIGH
BUN/CR RATIO	5-24 ratio	7	WNL
GFR	>60 m:/min	30	WNL

## Discussion

The role of COVID-19 in the development of hypothyroidism has not been well defined. Although hypothyroidism may develop from autoimmune processes, the SARS-CoV-2 virus currently has no direct linkage to the formation of Hashimoto's, subacute thyroiditis, or other related pathologies. Burekovic et al. highlighted 21 patients in the year 2020 and 29 in the year 2021 who developed hypothyroidism following COVID-19 (p < .01) [6]. However, the average time of diagnosis was two to three months following infections with COVID-19. The patient in our study was diagnosed with hypothyroidism in conjunction with COVID-19. Her official diagnosis was delayed due to her primary presentation in the emergency department. However, the patient demonstrated a positive COVID-19 test and highly elevated TSH levels, supporting the theory that her infection with COVID-19 prompted a hypothyroid state. Furthermore, there have been limited reports of subacute thyroiditis causing hypothyroidism in patients following resolution from the SARS-CoV-2 virus [7]. 

As previously mentioned, the pathogenesis of SARS-CoV-2 coronavirus host-cell entry provides an intriguing exploration of the relationship between thyroiditis and COVID-19. Recent case reports have outlined subacute thyroiditis as a common sequelae of COVID-19 in mostly females, but these studies revolve around symptoms of hyperthyroidism complicated by thyrotoxicosis. Our patient represents a minority of patients who experienced SARS-CoV-2 which presented with complications of hypothyroidism. A cohort observational study conducted by Khoo et al. followed 185 patients with COVID-19 using TSH and free T4 levels at baseline, hospital admission, and one-year follow-up. The observational study showed 86.6% of the patients were euthyroid at original admission, while only 0.6% of the patients were hypothyroid [8]. Unfortunately at one-year follow-up, the study sample size dropped to 55 patients due to COVID-19-related mortality. Of the remaining sample size, two patients developed secondary hypothyroidism and two patients had subclinical hypothyroidism [8]. The study showed that there is a link between thyroid dysfunction and COVID-19, but a pitfall to this study was the loss of hypothyroid patients prior to the one-year follow-up. Thus, adequate speculation into a relationship between acute-onset hypothyroidism and COVID-19 was not able to be explored. However, it has been demonstrated in recent literature that pre-existing thyroid disorders prior to viral infection have shown strong association with COVID-19 disease outcome [9]. 

The largest prospective study conducted to date by Lui et al. followed 122 COVID-19-surviving patients for 90 days to assess thyroid function and autoimmunity. Out of all patients examined only 20 patients were admitted with abnormal thyroid function test (TFT) [10]. Ultimately, patient 20 was the only patient that required levothyroxine treatment on follow-up assessment [10]. The authors rationalized the patient's outcome to a possible pre-existing Hashimoto’s thyroiditis, but did not consider the likelihood of COVID-19-induced hypothyroidism. Another pitfall to this study was the failure of further thyroid imaging other than TFT and anti-thyroglobulin titers for examination [10]. Additional prospective studies and future meta-analyses should be conducted to better understand a linkage of acute-onset hypothyroidism precipitated by COVID-19 infection as we progress from the pandemic and gain a better understanding of the infection. 

## Conclusions

We present a case of an elderly female with symptomatic bradycardia and positive COVID-19 PCR test. Laboratory studies revealed a hypothyroid state. Early detection and appropriate treatment with levothyroxine lead to prompt resolution of the patient’s bradycardia and dyspnea. Current literature showing a relation between COVID-19 and acute-onset hypothyroidism is scarce; our aim was that this case report can add to current literature when evaluating any possible causative relationships. Additionally, screening for TSH and free T4 levels in COVID-19 patients should be considered in appropriate circumstances to prevent the possible complications of hypothyroid cardiomyopathy or myxedema coma.
